# Immune Evasion and Drug Resistance Mediated by USP22 in Cancer: Novel Targets and Mechanisms

**DOI:** 10.3389/fimmu.2022.918314

**Published:** 2022-07-20

**Authors:** Jinhui Guo, Jie Zhao, Wen Fu, Qiuran Xu, Dongsheng Huang

**Affiliations:** ^1^ Qingdao Medical College, Qingdao University, Qingdao, China; ^2^ Laboratory of Tumor Molecular Diagnosis and Individualized Medicine of Zhejiang Province, Zhejiang Provincial People’s Hospital, Affiliated People’s Hospital, Hangzhou Medical College, Hangzhou, China; ^3^ College of Biotechnology and Bioengineering, Zhejiang University of Technology, Hangzhou, China

**Keywords:** ubiquitylation, deubiquitination, cancer, DUBs, USP22, immune evasion

## Abstract

Regulation of ubiquitination is involved in various processes in cancer occurrence and development, including cell cycle arrest, cell proliferation, apoptosis, invasion, metastasis, and immunity. Ubiquitination plays an important role not only at the transcriptional and post-translational levels but also at the protein level. When ubiquitination is in a pathological state, abnormally activated biological processes will not only induce cancer progression but also induce immune evasion. The main function of deubiquitinases (DUBs) is to remove ubiquitin chains from substrates, changing the biological activity of the substrates. It has great potential to improve the prognosis of cancer by targeting DUB to regulate proteome. Ubiquitin-specific peptidase 22 (USP22) belongs to the ubiquitin-specific protease (USP) family of DUBs and has been reported to be related to various physiological and pathological processes. USP22 is abnormally expressed in various malignant tumors such as prostate cancer, lung cancer, liver cancer, and colorectal cancer, which suggests that USP22 may play an important role in tumors. USP22 may stabilize programmed death ligand 1 (PD-L1) by deubiquitination while also regulating T-cell infiltration into tumors. Regulatory T cells (Tregs) are a unique class of immunosuppressive CD4^+^ T cells that primarily suppress the immune system by expressing the master transcription factor forkhead box protein 3 (FOXP3). USP22 was found to be a positive regulator of stable FOXP3 expression. Treg-specific ablation of USP22 leads to reduced tumor volume in multiple cancer models. This suggests that USP22 may regulate tumor resistance to immunotherapy. In this article, we review and summarize the biological functions of USP22 in multiple signal transduction pathways during tumorigenesis, immune evasion, and drug resistance. Furthermore, we propose a new possibility of combining USP22 with chemotherapeutic, targeted, and immunosuppressive drugs in the treatment of cancer.

## Introduction

**Figure 1:**
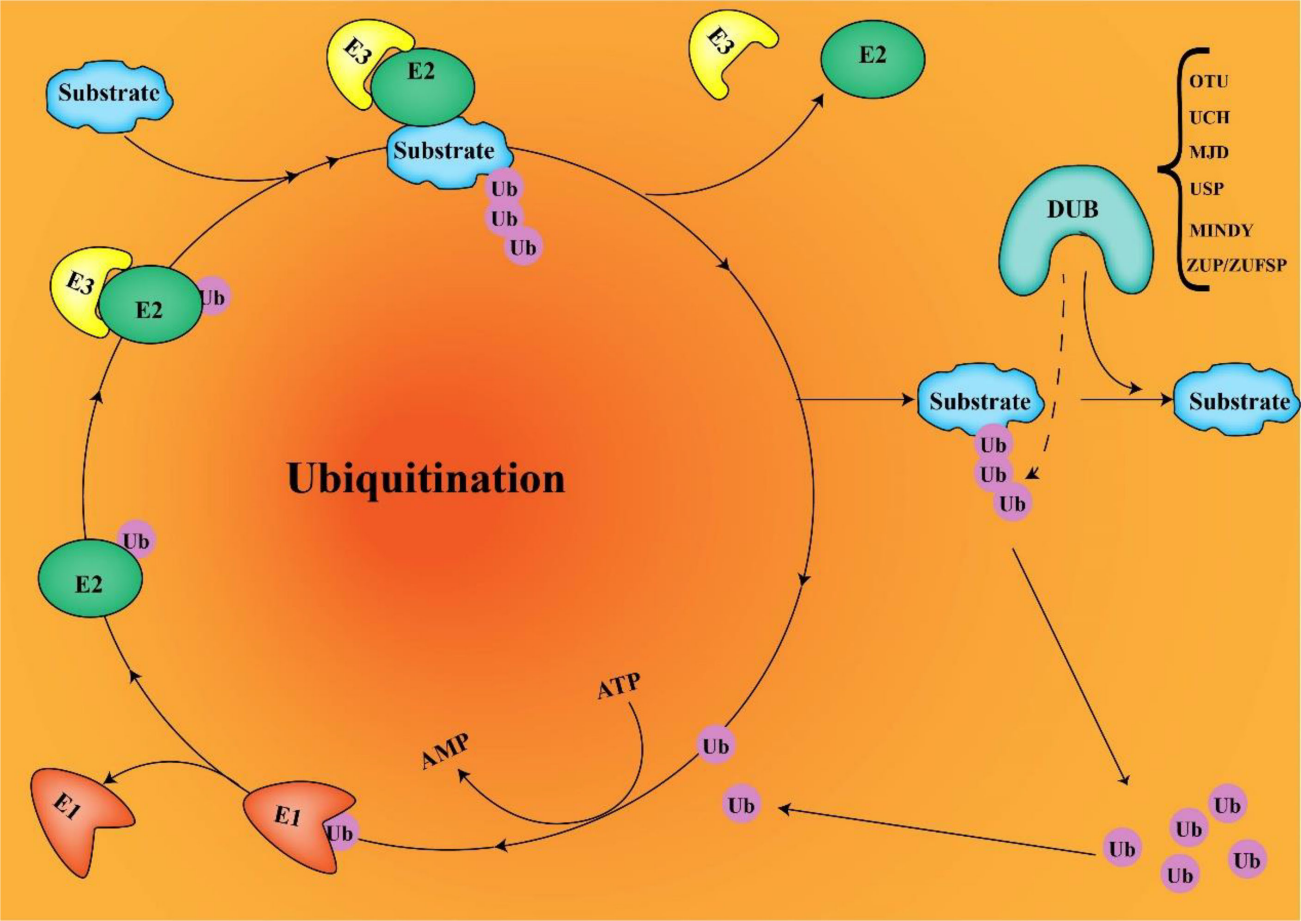
E1, E2, and E3 ubiquitination and DUB deubiquitination (ubiquitin connects to target proteins *via* E1, E2, and E3. DUB removes ubiquitin from substrates and recovers ubiquitin molecules).

Ubiquitination is an essential post-translational modification process in all cells that regulates protein activation/inactivation, DNA repair, gene regulation, and signal transduction (1, 2). Substrate proteins are covalently linked to ubiquitin through isopeptide bonds catalyzed by the E1-E2-E3 ligase cascade, mediating a range of biological effects *via* (3) (**Figure 1**). Ubiquitin can bind to target proteins as monoubiquitin or polyubiquitin. Monoubiquitination has functions such as endocytosis, DNA damage, and subcellular protein localization. Polyubiquitination enables physiological activities such as protein degradation. Ubiquitin is a small protein composed of 76 amino acids, containing seven lysine sites (K6, K11, K27, K33, K48, and K63), a methionine site (M1) at the N-terminus, and a C-terminal glycine site (G76). Ubiquitins are mainly connected by lysine residues (K6, K11, K27, K29, K33, K48, and K63) and methionine residues (M1). The K48 chain and K63 chain are the most studied ubiquitin chain linkages that guide the expression of substrate proteins. The K48 ubiquitin chain has been shown to play an important role in ATP-dependent proteasomal degradation (4), whereas the K63 ubiquitin chain is mainly involved in the modification of protein location and function (5). Deubiquitinases (DUBs) regulate a variety of cellular functions by removing ubiquitin chains from substrates. The ubiquitination process requires three enzymes. However, DUBs are a single enzyme that antagonizes not only the ubiquitination of substrate proteins but also the autoubiquitination of E3 ligases.

With numerous studies on the role of various DUBs in cancer, DUBs are becoming attractive therapeutic targets because they control important biological processes and are easily controlled by drugs. Currently, more than 100 DUBs have been found in humans, divided into six different families according to their structure and function (6): ubiquitin-specific proteases (USPs), ovarian tumor proteases, ubiquitin C-terminal hydrolases, Machado-Joseph disease protein proteases, the motif interacting with ubiquitin–containing novel DUB family, and Zinc Finger USP. USPs are the largest and most diverse group of DUBs, accounting for about 60% (7). Members of the USPs family are highly conserved and contain three subdomains similar to the right hand finger, thumb, and palm (8). Upregulated USP22 develops resistance to conventional therapy and increases the risk of cancer cell metastasis, resulting in patient death (9, 10). In many types of tumors, USP22 was identified with a oncoprotein and was in abundance with tumor progression (11–13). USP22 is overexpressed in a variety of tumors and plays a role in promoting cancer. Further understanding of the regulatory mechanism of USP22 expression may help to improve anti-tumor therapy.

USP22 is a key subunit of the Spt-Ada-Gcn5 acetyltransferase complex (SAGA) that removes ubiquitin from target proteins, thereby regulating transcription of downstream genes (14). Many transcription factors are controlled by the SAGA complex during cancer progression, including the androgen receptor (AR), the oncogene c-MYC, and the tumor suppressor P53 (9). USP22 induces changes in gene promoter regions by deubiquitinating histones H2A and H2B, thereby controlling transcription. USP22 can be considered a broad transcriptional activator that regulates multiple proteins. MYC is a proto-oncogene that plays an important role in regulating tumor invasion, and the transcriptional activity of MYC and its target genes requires the mediation of USP22 (15). USP22 inhibits activation of apoptotic pathways by stabilizing SIRT1. USP22 is also closely related to the cell cycle. USP22 directly deubiquitinates cyclin D1, protects it from protease-mediated degradation, and promotes tumor proliferation *in vivo* (16).

Given the important role of USP22 in tumor progression, USP22 may become a new potential target for tumor therapy. Although treatments for cancer [chemotherapy, immune checkpoint inhibitors (ICIs), and targeted drugs] have shown some benefit, most patients develop resistance. Therefore, there is an urgent need to find new targets to overcome the drug resistance problem. Elucidating the mechanism of USP22 in cancer drug resistance will help to break through the predicament of cancer multidrug resistance in clinical practice.

## Cancer Immune Evasion

Cancer immunotherapy has made great clinical progress in the past few years, such as pembrolizumab and nivolumab for melanoma and non–small cell lung cancer (NSCLC). Programmed death ligand 1 (PD-1) belongs to the B7 family and is a 33-kDa type 1 transmembrane glycoprotein containing 290 amino acids. PD-L1, a ligand for PD-1, is commonly expressed by macrophages, activated T cells, B cells, and dendritic cells (especially under inflammatory conditions). Furthermore, PD-L1 is expressed by tumor cells as an “adaptive immune mechanism” to evade anti-tumor responses. The PD-1/PD-L1 axis negatively regulates immune responses by inhibiting T-cell activation, proliferation, and attenuating CD8^+^ T cells in the tumor microenvironment. Conversely, blocking the PD-1/PD-L1 pathway activates cytotoxic T-cell responses that specifically kill tumor cells using ICIs. Except for bladder cancer, melanoma, and some special blood diseases, the overall response rate of targeting PD-1/PD-L1 is still generally low, so an in-depth understanding of the resistance mechanisms of ICIs is required. Recent studies have shown that the clinical efficacy of anti–PD-1/PD-L1 axis drugs correlates with PD-L1 levels (17). Cancer cells use multiple mechanisms to achieve immune evasion, including upregulation of the immune checkpoint ligand PD-L1 and inhibition of antigen presentation mechanisms.

According to the presence or absence of tumor-infiltrating lymphocytes, tumors are classified into “cold tumors” and “hot tumors”. “Hot” and “cold” reflect whether the tumor is immunogenic to respond to immunotherapy. The abundance of tumor-infiltrating T cells is a major predictor of immunotherapy response, as T-cell–infiltrating tumors are more sensitive to ICIs than non–T-cell–infiltrating tumors. Regulation of USP22 can alter the tumor microenvironment, thereby turning cells that are completely resistant to immunotherapy into a sensitive state. In pancreatic ductal adenocarcinoma (PDAC), knockdown of USP22 demonstrated better response to immunotherapy, with increased proportion of natural killer (NK) cells and CD8^+^ T cells in the tumor (18). Similar results have been reported in liver tumors (19), where ablation of USP22 in liver tumor cells has been shown to increase tumor immunogenicity and promote T-cell infiltration into the resulting liver tumors. The expression of USP22 in tumor cells suppresses anti-tumor immunity and confers resistance to immunotherapy (18).

Understanding the regulation of PD-L1 expression may help improve anti–PD-L1/PD-1 therapy. PD-1/PD-L1 expression is regulated by multiple pathways. Phosphatidylinositol 3-Kinase (PI3K)/AKT pathway, mitogen-activated protein kinases (MAPK) pathway, janus kinase/ signal transducer and activator of transcription (JAK/STAT) pathway, Wingless and int-1 (WNT) pathway and nuclear factor kB (NF-kB) pathway can all promote the expression of PD-1/PD-L1 axis. Furthermore, oncogenic RAS signaling can stabilize PD-L1 mRNA to promote tumor immune resistance (20). Post-translational modifications (ubiquitination, glycosylation, methylation, and phosphorylation) of PD-L1 play an important role in immune inactivation and suppression (21, 22). Furthermore, increasing evidence suggests that the ubiquitin-proteasome system–mediated regulation of PD-L1 stability directly affects the efficacy of anti–PD-1/PD-L1 treatments.

**Figure 2:**
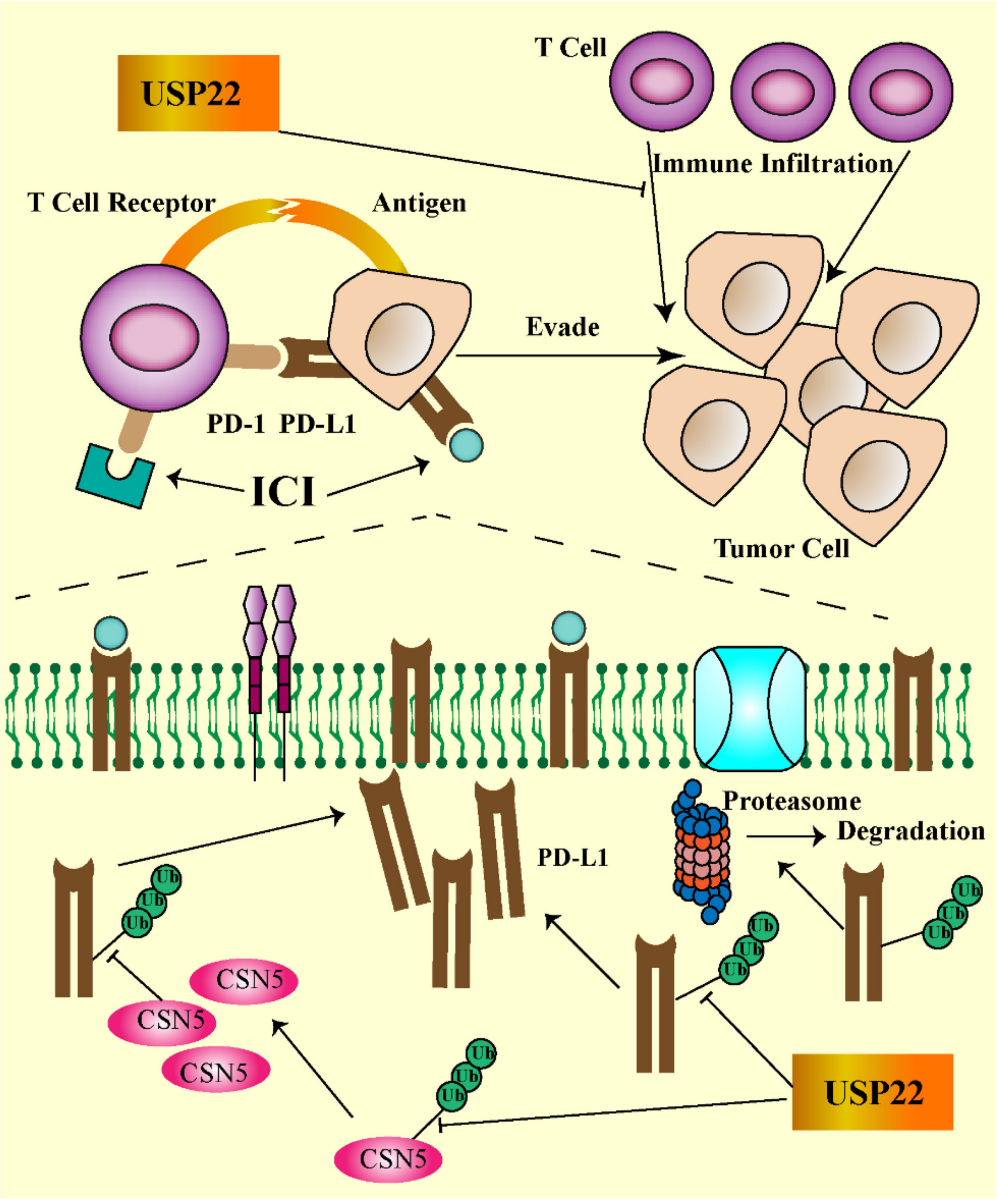
USP22-mediated immune evasion (USP22 can directly regulate PD-L1 stability through deubiquitination; on the other hand, USP22 regulates PD-L1 protein levels through the USP22-CSN5-PD-L1 axis. USP22 expression inhibits T-cell infiltration into tumors. The PD-1/PD-L1 axis contributes to tumor cell escape by inhibiting T-cell activation).

In normal tissues, USP22 are involved in T- and B-cell growth, development, and phenotype switching. In cancer, USP22 may alter the immune microenvironment. Loss of USP22 not only enhances the sensitivity of cisplatin-based chemotherapy but also improves the efficacy of PD-L1–targeted immunotherapy (19). This suggests that USP22 may be a powerful target for resistance to PD-L1/PD-1 blockade therapy. Replacing lysine with arginine in the intercellular domain of PD-L1 prevents USP22 depletion-induced downregulation of PD-L1, suggesting that USP22 stabilizes PD-L1 through deubiquitination of lysine (23). USP22 regulates PD-L1 degradation in two ways (**Figure 2**). On the one hand, USP22 can directly regulate the stability of PD-L1 through deubiquitination, leading to tumor immune resistance. On the other hand, USP22 deubiquitinates CSN5 and regulates PD-L1 protein levels through the USP22-CSN5-PD-L1 axis. CSN5 is required for PD-L1 stabilization in cancer cells and was identified as a key protein required to promote PD-L1 deubiquitination. USP22 can remove polyubiquitin chains of CSN5 and stabilize CSN5 protein through its deubiquitination activity. Considering that USP22 can increase CSN5 protein levels, there may be a positive feedback mechanism. USP22 enhances the stability of CSN5, which, in turn, promotes the interaction between USP22 and PD-L1, suggesting that USP22 and CSN5 synergistically regulate PD-L1.

It should be noted that USP22 plays a positive regulatory role in certain T-cell subsets. Invariant NK T (iNKT) cells play a key role in the immune system and are implicated in autoimmunity and tumor surveillance. USP22 is essential for iNKT development, and loss of USP22 function prevents iNKT cell development (24). In addition, USP22 may also play an important role in the activation of T cells. USP22 can promote the expression of interleukin-2 in T cells by deubiquitinating and stabilizing NFATc2 (an important regulator of T-cell activation)—a novel role for USP22 as a positive regulator of NFATc2 in the control of T-cell immune responses (25).

## Resistance Mechanism of USP22

### Hypoxic Microenvironment

**Figure 3:**
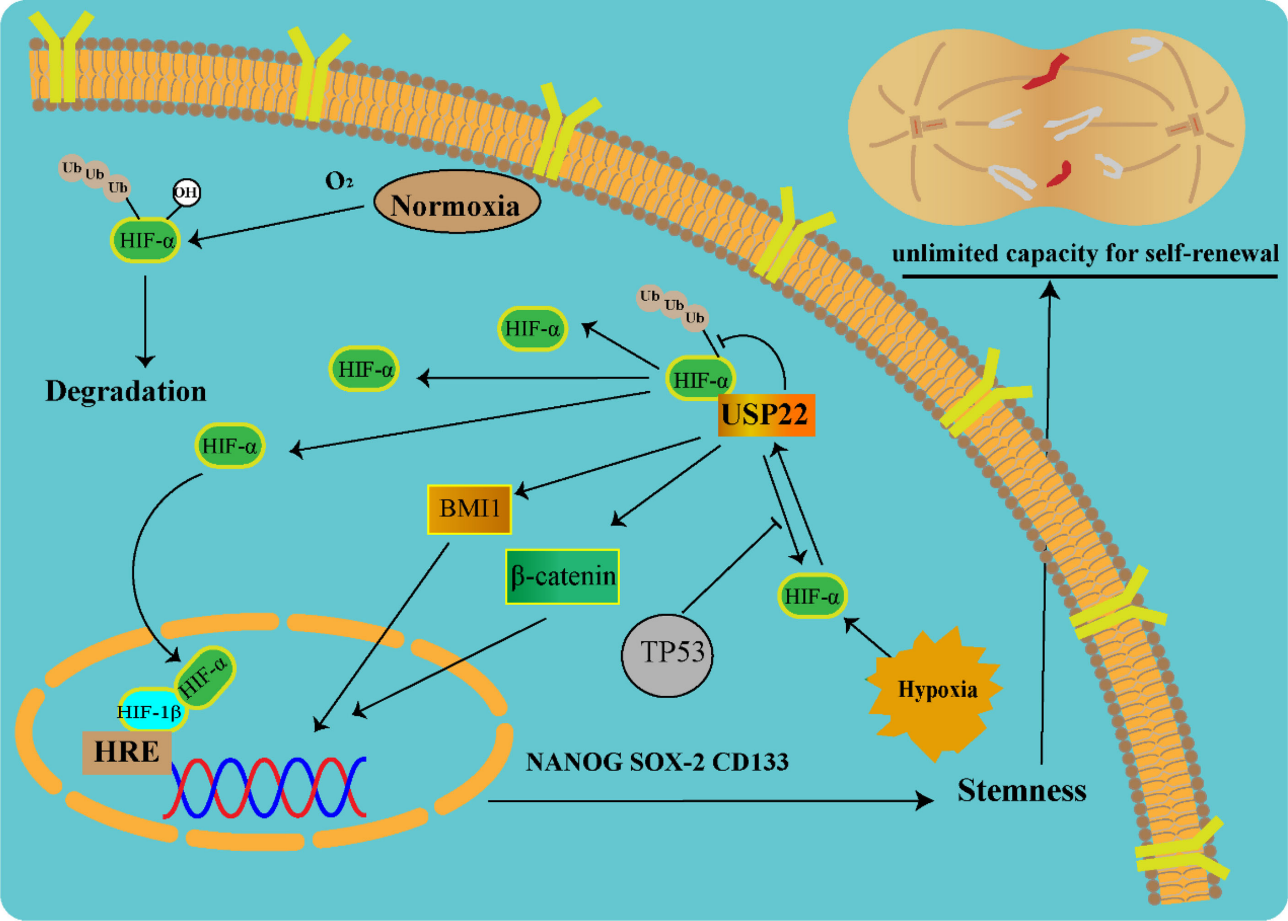
USP22-mediated tumor stemness in a hypoxic microenvironment (USP22 upregulates stemness gene expression *via* Wnt/β-catenin, HIF-1α, and BMI1 pathways).

Hypoxia is one of the most important features of the tumor microenvironment and plays a key role in the maintenance of tumor stem cells. Solid tumor microvascular abnormalities and uninhibited growth are prone to hypoxic microenvironment (26). Once tumors adapt to the hypoxic microenvironment, surviving tumor cells become more malignant and become resistant to chemotherapy. Under hypoxic conditions, hypoxia-inducible factor (HIF) can regulate downstream gene expression and promote tumor malignant progression (27). HIF consists of a stable HIF-1β subunit and an oxygen-sensitive α subunit (HIF-1α, HIF-2α, and HIF-3α). Under normoxic conditions, HIF-1α is degraded *via* the ubiquitin-proteasome pathway (28). Under hypoxic conditions, HIF-1α was stably transcribed and combined with HIF-1β to promote the transcription of downstream stemness genes (NANOG, SOX-2, and CD133). USP22 enhances the stability and transcriptional activity of HIF-1α under hypoxia through deubiquitination and induces upregulation of HIF-1α downstream genes. In TP53-mutated hepatocellular carcinoma, USP22 and HIF-1α promote the stabilization of each other, forming a positive feedback loop (29). In TP53 wild-type hepatocellular carcinoma, HIF-1α directly promotes the transcription of TP53 gene, and the protein of TP53 gene, in turn, blocks the positive regulation of USP22 by HIF-1α (**Figure 3**).

Cancer stem cells (CSCs) are a subset of cells capable of self-renewal, unlimited replication, and multidirectional differentiation. CSCs are able to survive in a hostile tumor microenvironment, making them a resistant subset of cancer cells. Anti-tumor therapies such as conventional chemotherapy and molecularly targeted therapy are often ineffective for CSC treatment and lead to CSC enrichment (30). Mechanisms such as apoptosis inhibition, protective autophagy, cell cycle acceleration and DNA repair, and epithelial–mesenchymal transition (EMT) are hyperactive in CSCs. Therefore, reducing the stemness of tumors may also reduce their chemoresistance (31). Further studies found that USP22 is not only a CSC marker but also upregulated after drug resistance in multiple cancers.

B-cell–specific Moloney murine leukemia virus integration site 1 (BMI1) is a key regulatory component of polycomb repressive complex 1 involved in CSC self-renewal and maintenance of CSC stemness (32). USP22 and BMI1 form a multiprotein complex acting on their target homologous (Hox) gene clusters. Hox genes play a central role in the directed differentiation and proliferation of cells. USP22-BMI1 silences the Hox gene, thereby increasing tumor resistance (33). In hypoxic microenvironment, HIF-1α accelerates glioma cell stemness, invasion, and metastasis by regulating the USP22-BMI1 axis (34). In gastric cancer, USP22 maintains CSC stemness by stabilizing BMI1 protein. The expressions of USP22 and BMI1 are closely related in various cancer tissues, such as liver cancer, colorectal cancer, and gastric cancer. Co-expression of USP22 and BMI1 can accelerate tumor proliferation, stemness, and drug resistance (35).

Activation of HIF mediates angiogenesis, stem cell maintenance, invasion, metastasis, and resistance to chemoradiotherapy under tumor hypoxia. Knockout of HIF-1α attenuated GSC stemness gene expression, inhibited cell growth, and promoted cell apoptosis. USP22 silencing significantly downregulated BMI1 protein expression and further affected gastric CSC self-renewal. Through clinical specimen analysis, overexpression of USP22 and BMI1 was associated with gastric cancer progression and treatment failure (36). In addition, USP22 promotes CSC maintenance through the Wnt/β-catenin pathway (37). Because it is currently difficult to use HIF-1α as a direct drug target, the indirect intervention of HIF-1α through USP22 is considered a new research idea.

### 5-Fluorouracil

5-Fluorouracil (5-FU), a pyrimidine analog, belongs to the drug family of antimetabolites and is widely used in clinical practice. The anticancer mechanism of 5-FU is mainly through the non-competitive inhibition of thymidylate synthase (TS), which is then synthesized into RNA and DNA, thereby inducing cytotoxicity (38). As a pyrimidine analog, it can be misincorporated into RNA and DNA in place of uracil or thymine and then converted to different cytotoxic metabolites. 5-FU causes DNA and RNA dysfunction by interfering with nucleoside metabolism, inducing cell cycle arrest and apoptosis (39). Despite the many advantages of 5-FU, its clinical application has been greatly limited due to drug resistance. Approximately 50%–60% of colorectal cancer patients will eventually develop resistance to 5-FU, resulting in poor survival outcomes (40). Research shows that inhibition of USP22 can increase the sensitivity of hepatoma cells to 5-FU.

Sirtuin 1 (SIRT1) is a member of the sirtuin family of nicotinamide adenine dinucleotide (NAD^+^)–dependent class III histone deacetylases (41). SIRT1 is a mediator of acetylation of the USP22 and SAGA coactivator complex. SIRT1 was previously reported to be an important mediator of USP22-driven cancer resistance, promotes HCC cell proliferation, and enhances resistance to chemotherapy (42). USP22 directly interacts with SIRT1 and then activates AKT/GSK-3β/MRP1, which, in turn, promotes chemotherapeutic efflux in HCC cells (43). C-MYC and SIRT1 form a positive feedback loop in the cell, increasing each other’s stability. USP22 increases MYC-mediated SIRT1 protein stability. USP22 reduces P53 levels by stabilizing SIRT1, thereby inhibiting apoptosis during DNA damage and embryonic development (44). Unlike USP14, USP22 still has no effect-specific small-molecule inhibitor. However some non-coding RNAs can regulate the activity of USP22. Studies have shown that the tumor suppressor miR-4490 can bind to sequences within the 3′-UTR of USP22 and inhibit the expression of USP22 in gastric cancer cells. This study may provide new ideas for inhibiting the expression of USP22. In addition, USP22 may induce autophagy through deubiquitination of SIRT1, thereby reducing the sensitivity of hepatoma cells to chemotherapeutic drugs (45).

In colorectal cancer, USP22 may induce chemoresistance through the Wnt/β-catenin signaling pathway. Mechanistically, there is evidence that USP22 promotes cell cycle progression by increasing β-catenin nuclear localization, which is required for Wnt pathway activation. The Wnt/β-catenin pathway is an evolutionarily conserved signal transducer responsible for regulating many normal physiological processes, such as cell proliferation, cell differentiation, and cell polarity. Abnormal activation of Wnt/β-catenin signaling is closely associated with increased prevalence and malignant progression (46, 47). In addition, the Wnt/β-catenin pathway is one of the important oncogenic pathways associated with immune escape. In colorectal cancer, the Wnt/β-catenin pathway promotes CSC maintenance, tumorigenesis, and chemoresistance (48). MiR-30-5p attenuates the Wnt/β-catenin pathway by targeting down the expression of USP22, thereby negatively regulating CRC stemness and chemoresistance. Drug resistance suppressed by miR-30-5p in colorectal cancer cells is partially abolished by USP22 overexpression (37). In addition, studies have also shown that p53 is a key regulator of 5-FU chemoresistance induced by colorectal cancer cells through the WNT/β-catenin signaling pathway (49). This suggests that USP22/Wnt/β-catenin signaling can mediate 5-FU resistance in colorectal cancer cells.

**Figure 4:**
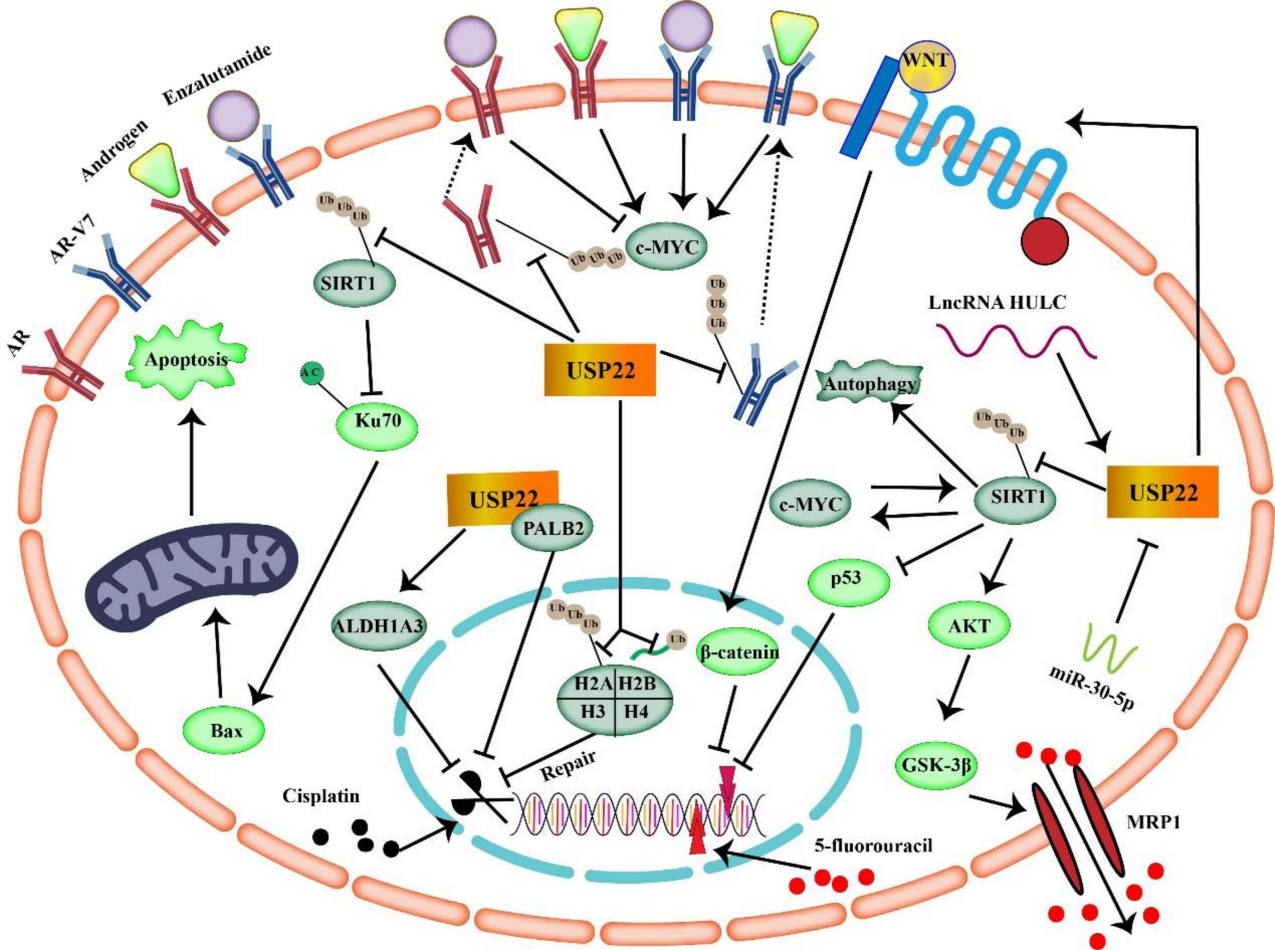
USP22 mediates tumor resistance mechanisms to enzalutamide, cisplatin, and 5-fluorouracil (USP22 can make cancer cells resistant to 5-FU through SIRT1 and Wnt/β-catenin signaling. USP22 mediates tumor resistance to cisplatin by acting on ALDH1A3, PALB2, H2A, H2B, and SIRT1. Binding of USP22 to AR/AR-V7 prevents AR/AR-V7 protein degradation and enhances tumor resistance to enzalutamide).

Growing evidence suggests that the main cause of cancer cell resistance to 5-FU is increased stemness characteristics (50, 51). Because traditional chemotherapy targets actively proliferating cancer cells, quiescent CSC populations take the opportunity to lie dormant, causing cancer to recur at a later stage. In addition, CSCs can reprogram their signaling pathways to adapt to environmental changes in harsh environments, which is essential for them to maintain their ability to proliferate indefinitely. The resistance of CSCs to 5-FU can be attributed to aberrant activation of different growth signaling pathways and resistance to DNA damage. It was found that 5-FU resistant cells led to the upregulation of stem cell markers (CD44, OCT4, SOX2, and NANOG) and enhanced the ability of tumor spheroid formation, cloning, migration, and invasion (51) (**Figure 4**).

In conclusion, USP22 is upregulated in a variety of malignancies, and studies have shown that USP22 induces tumor resistance to 5-FU by acting on CSCs, SIRT1, and C-MYC. In addition, upregulation of USP22 in tumors often implies poor prognosis. Therefore, targeting USP22 in 5-FU–resistant tumors may help improve efficacy.

### Cisplatin

Cisplatin is widely used in the treatment of many types of solid tumors and has become the gold standard for the treatment of many cancers (52). It is the first platinum compound approved by the FDA for cancer treatment in 1978 and has a strong broad-spectrum anticancer effect. Cisplatin generates intra- and inter-strand crosslinks by binding to DNA, resulting in DNA damage and subsequent induction of cancer cell cycle arrest and apoptosis (53). Unfortunately, most patients develop resistance to cisplatin therapy, ultimately leading to treatment failure and tumor progression. Cisplatin resistance depends on multiple factors such as induction of anti-apoptotic signals, drug inactivation, increased DNA repair, increased stemness, and EMT (54). Unlike 5-FU resistance mechanisms, USP22 increases tumor resistance to cisplatin by enhancing DNA repair capacity. Homologous recombination repairs DNA double-strand breaks through a template-dependent mechanism to maintain genome integrity. PALB2 protein is a key protein in homologous recombination repair. Cisplatin kills cancer cells by causing double breaks in the cell’s DNA. USP22 directly interacts with PALB2 *via* the C-terminal WD40 domain to promote DNA homologous recombination repair (55).

Cytotoxic chemotherapy drugs (such as cisplatin) are the first-line drugs for the treatment of NSCLC, and platinum-based chemotherapy resistance is one of the important reasons for the failure of treatment of advanced NSCLC. In the study of the correlation between USP22 and acquired cisplatin resistance in lung adenocarcinoma, the expression of USP22 was found to be upregulated in cisplatin-resistant lung adenocarcinoma cells (56). The overexpression of USP22 resulted in the resistance of lung cancer cells to cisplatin. USP22 promotes phosphorylation of histone H2AX by deubiquitinating histone H2A, enhancing DNA damage repair, and inducing cisplatin resistance (56). Loss of histone H2B monoubiquitination (H2Bub1) is associated with poor differentiation, cancer stemness, and chemotherapy resistance in NSCLC. H2Bub1 levels are regulated by the inverse activity of the DUB USP22 (57). USP22 inhibition restores cisplatin sensitivity in cisplatin-resistant lung cancer cells.

Cisplatin can induce cell death by activating mitochondrial apoptosis. USP22 upregulation can overcome cisplatin-induced cycle arrest and inhibit apoptosis (56). Bax is one of the core pro-apoptotic proteins of the intrinsic mitochondrial apoptotic pathway. Deletion of Bax resulted in complete resistance to cisplatin. An important mechanism of apoptosis induction is the activation of Bax protein. In normal cells, Ku70 is a DNA repair protein that sequesters. Bax proteins in the cytoplasm to prevent the initiation of apoptosis and prolong cell survival. Lysine residues in the Ku70 domain are acetylated, resulting in Bax release (58). USP22 can reduce Ku70 acetylation by stabilizing SIRT1 expression, thereby inhibiting Bax-mediated apoptosis and promoting cisplatin resistance.

ALDH1A3, a major ALDH isoenzyme, is important for the stem cell signature of lung cancer and is associated with enhanced cisplatin resistance in lung adenocarcinoma. After knockdown of USP22, ALDH1A3 was significantly downregulated in tumor cells. Knockdown of ALDH1A3 sensitizes tumor cells to cisplatin (57). Lung adenocarcinoma cells may resist the cytotoxicity of cisplatin by enhancing ALDH activity. USP22 regulates ALDH activity by transcriptionally regulating ALDH1A3 levels.

Enhanced DNA repair processes, increased cell stemness characteristics, and inhibition of apoptotic pathways may be major factors in USP22-induced cisplatin resistance. USP22 directly interacts with PALB2 to promote DNA homologous recombination repair and inhibit the killing effect of cisplatin on cancer cells. In addition, USP22 can also enhance DNA damage repair by deubiquitinating histones H2A and H2Bub1. Targeting CSCs is a promising direction for developing new tumor therapies. Downregulation of USP22 significantly impairs lung cancer stemness and resistance to cisplatin. Overall, downregulation of USP22 is expected to enhance the therapeutic effect of cisplatin in tumors.

### Androgen Receptor

The AR, a steroid receptor transcription factor for testosterone and dihydrotestosterone, plays an important role in the development and progression of prostate cancer and is a key therapeutic target (59, 60). Androgens act as ligands that bind to AR, and the activated AR binds to the DNA sequences of downstream genes and initiates the expression of a series of genes that promote prostate cancer progression. Enzalutamide is a next-generation AR pathway inhibitor that binds to the ligand-binding domain of AR and disrupts the interaction between AR and androgen. Androgen deprivation therapy refers to the reduction of androgen levels in the body through various means and is the main treatment method for patients with advanced prostate cancer. Unfortunately, prostate cancer can alter AR during treatment, into incurable and lethal prostate cancer (61, 62). Patients with hormone-sensitive prostate cancer initially respond well, but they tend to develop resistance to this therapy in most cases. Prostate cancer that is resistant to androgen deprivation therapy is called castration-resistant prostate cancer (CRPC).

USP22 controls AR accumulation and signaling, and it enhances the expression of key target genes co-regulated by AR and MYC (63). USP22 not only reprograms AR function but also transforms tumors toward therapy resistance. In prostate cancer, elevated MYC drives tumorigenesis, and MYC gene activation has been shown to correlate with cancer progression and poor survival (64). MYC is downstream of AR and promotes prostate cancer cell growth even in the absence of androgens. USP22 is a functional mediator necessary for MYC to promote cancer and can increase the stability and tumorigenic activity of MYC in cancer cells (65, 66). Overexpression of USP22 enhanced AR protein accumulation, which, in turn, activated downstream target genes regulated by AR and MYC. This USP22-mediated activation can bypass androgens or AR antagonists (enzalutamide) to induce castration resistance in prostate cancer.

AR splice variant 7 (AR-V7), a ligand-independent activating variant of the AR, is thought to be a key driver of CRPC (67). Targeted AR therapy is limited in CRPC due to lack of ligand-binding domain of AR-V7 (68). Among the AR splice variants (AR-Vs), AR-V7 is the most abundant variant and has the highest detection frequency in prostate cancer. It should be noted that AR-V7 is the only endogenous variant detected at the protein level and can show functional activity in the absence of androgens (69, 70). AR-V7 is 20-fold higher in CRPC compared to hormone-naive prostate cancer (71).

Nobiletin is a natural product extracted from the peel and belongs to the polymethoxyflavonoids (72).Nobiletin mediates proteasomal degradation of AR-V7 by promoting ubiquitination of AR-V7 protein in CRPC cells (72). USP22 acts as a deubiquitinating enzyme that regulates the stability of AR-V7 protein. Protein analysis showed that USP22 reduction significantly reduced the half-life of AR-V7. Conversely, overexpression of USP22 slowed down AR-V7 degradation to some extent, partially enhancing the viability of CRPC cells. Collectively, nobiletin selectively induces AR-V7 degradation by inhibiting the interaction of AR-V7 with USP22.

Prostate cancer relies on AR signaling in both disease initiation and progression. Although various therapeutic options are used to inhibit AR signaling, the reactivation of AR signaling in CRPC cells is still difficult to control. USP22 is a novel driver of CRPC progression by regulating AR protein accumulation. AR-V7 can activate the AR pathway in the absence of androgens, which is the biggest challenge in the treatment of CRPC. Binding of USP22 to AR-V7 prevents AR-V7 protein degradation. Targeting USP22 can resist enzalutamide resistance, which may provide a new perspective for the treatment of CRPC.

## USP22 Is a New Therapeutic Target for Cancer

### Lung Cancer

Lung cancer continues to have a high incidence and is the leading cause of cancer death worldwide (73). The 5-year survival rate for stage I patients is approximately 80%, and the 5-year survival rate for stage II–III patients is 13%–60% (74). NSCLC is the most common type of lung cancer, accounting for 85% of all cases. According to histological classification, NSCLC can be divided into adenocarcinoma, squamous cell carcinoma, and large cell carcinoma.

Elevated USP22 in lung cancer predicts highly malignant clinical behavior and is associated with poorer overall survival (75). USP22 is highly expressed in lung adenocarcinomas compared to normal mucosa. The mechanism by which USP22 promotes NSCLC tumorigenesis is that USP22 can directly bind and upregulate MDMX (E3 ubiquitin ligase) in NSCLC cells and subsequently inhibit the P53 pathway to promote NSCLC tumorigenesis (76) (**Table 1**). EMT is considered to be a core mechanism of invasion and metastasis in various cancers, and overexpression of USP22 is able to regulate EMT to promote tumor progression in lung cancer (90).

**Table 1:** USP22 as a target for cancer.

Tumor	Target	First Author/s	References
NSCLC	USP22/MDMX/P53	Fangbao Ding	(76)
	USP22/EGFR/STAT3, AKT/mTOR, and MEK/ERK	Huijuan Zhang	(77)
	USP22/BMI1/Stemness	Jing Hu	(78)
	USP22/STAT1/T cell and NK cell	Bing Han	(75)
COLORECTAL CANCER	USP22/AP4/EMT	Rene Jackstadt	(79)
	USP22/BMI1/Akt and INK4a/ARF	Yan-Long Liu	(80)
	USP22/Wnt/β-catenin/Stemness	Shixiong Jiang	(81)
	USP22/CCND1/G1-S	Victoria J. Gennaro	(16)
	SNHG16/miR-132-3p/USP22	Xiaowen He	(82)
LIVER CANCER	USP22/Survivin/Apoptosis	Bo Tang	(83)
	USP22-E2F6-DUSP1-AKT	Tiantian Jing	(84)
	lncRNA HULC/USP22/COX-2	Haojun Xiong	(85)
BREAST CACER	USP22/c-Myc	Dongyeon Kim	(65)
	USP22/ERα	Shengli Wang	(86)
GLIOBLASTOMA	USP22/KDM1A	Aidong Zhou	(87)
GASTRIC CANCER	USP22/c-Myc/NAMPT/SIRT1/FOXO1 and YAP	Hongxia Liu	(66)
PDAC	USP22/DYRK1A	Zhile Bai	(88)
RETINOBLASTOMA	USP22/TERT/P53	D. Zhou	(89)
PROSTATE CANCER	USP22/MYC AR/CRPC	Randy S. Schrecengost	(63)

Activation of epidermal growth factor receptor (EGFR) tyrosine kinases can promote EMT and inhibit apoptosis in lung adenocarcinoma (77). Activating mutations in the EGFR gene are prevalent oncogenic factors in patients with NSCLC. USP22 prevents ubiquitination-mediated EGFR degradation, thereby inducing persistent activation of EGFR-mediated oncogenic signaling pathways, such as STAT3, AKT/mTOR, and MEK/ERK pathways. Important drug for the treatment of patients with EGFR-mutated lung adenocarcinoma is EGFR-tyrosine kinase inhibitors (TKI). USP22 not only enhances EGFR signaling activity but also promotes resistance to EGFR-TKIs.

BMI1 protein is a transcriptional repressor with the ability to maintain tissue-specific stem cell self-renewal. USP22 promotes stem cell-like features of NSCLC cells by regulating BMI1 signaling (78). In addition, the role of USP22 in the anti-tumor immunity of NSCLC has attracted more and more attention. Knockdown of USP22 can activate STAT1 signaling pathway, inhibit T-cell depletion, and promote the proliferation and activation of NK cells (75). In conclusion, USP22 plays an oncogenic role in lung cancer and may be an important target for the carcinogenesis and drug resistance mechanism of NSCLC. In prostate cancer, USP22 predicts disease outcome and promotes the CRPC phenotype by controlling AR and MYC dual regulation (63).

### Colorectal Cancer

Colorectal cancer (CRC) has the third highest incidence and is one of the leading causes of cancer-related deaths worldwide (91). To make matters worse, metastatic cases account for about 40% to 50% of colorectal cancer cases, and its OS is only 30 months (92). Both mRNA and protein levels of USP22 were expressed at higher levels in CRC tissues than in surrounding normal tissues, suggesting that USP22 regulation occurs not only at the protein level but also at the transcriptional level (93). The expression of USP22 increased from normal mucosa to colorectal cancer and was also significantly increased from adenoma to colorectal cancer. Interestingly, the expression of USP22 was not significantly upregulated from normal tissues to adenomas, suggesting that USP22 activation is enhanced during colorectal carcinogenesis. By analyzing 192 colorectal cancer patients, it was found that the expression of USP22 was associated with the occurrence of CRC metastasis and the increased chance of chemotherapy resistance.

Activating protein 4 (AP4) is a helix-loop-helix and leucine-zipper transcription factor that enhances CRC cell proliferation and invasion (79). USP22 increases AP4 transcription to induce EMT in colorectal cancer, which may induce tumor metastasis (94). BMI1, as a substrate of USP22, also has an important role in the colorectum. USP22 acts as an oncogene in colorectal cancer by activating BMI1, which, in turn, activates the INK4a/ARF pathway and Akt signaling pathway (80). In addition, multiple studies have shown (37, 81, 95) that USP22 can promote colorectal cancer stemness through the Wnt/β-catenin pathway. USP22 plays an important role in the progression of colorectal cancer by interfering with cell cycle progression. USP22 deubiquitinates G1 cyclin (CCND1), protecting it from proteasome-mediated degradation. CCND1 accumulation promotes G1-S transition in colorectal cancer cells (16). MiR-132-3p is a tumor suppressor gene in CRC and can inhibit the expression of USP22 (82). Overexpression of USP22 restored the inhibitory effect of miR-132-3p on CRC cell proliferation and metastasis. These studies provide strong evidence for USP22 as a molecular drug target for colorectal cancer. It should be noted that USP22 may have a bidirectional function in colorectal cancer. Studies have found that USP22 plays a tumor suppressor function in colorectal cancer by reducing mTOR activity (96).

### Liver Cancer

Liver cancer is one of the most common cancers in the world, and its incidence has been increasing in recent years, and it is the third leading cause of cancer death in the world (97). Because of the insignificant symptoms in the early stage, most patients with liver cancer are already in the middle and advanced stages when they are diagnosed. Even if radical surgical resection is performed, the survival rate of patients is only 25%–30%, and the recurrence/metastasis rate is 50%–70% (98–100). Hepatocellular carcinoma is the most common type of liver cancer. USP22 is not only expressed at elevated levels in hepatocellular carcinoma but also closely related to the malignant behavior of tumors. Kaplan–Meier analysis shows that elevated USP22 expression predicts poorer prognosis in patients (101).

Survivin is a member of the inhibitor of apoptosis protein family and is considered to be an inhibitor of apoptosis. The level of survivin is associated with poor prognosis of liver cancer. Increase in survivin may be regulated by translation and transcription of USP22 (83). USP22 may participate in HCC progression in cooperation with survivin. E2F6, an atypical member of the E2F family, is a transcriptional repressor (102). E2F6 can directly bind to the promoter region of dual-specificity protein phosphatase 1 (DUSP1) and repress its transcription. DUSP1 (an anti-apoptotic phosphatase) functions as a tumor suppressor in hepatoma cells and is also a negative regulator of AKT activation (103). E2F6 is essential for HCC cell growth, and its activity is controlled by USP22-mediated deubiquitination. USP22 removes the K48-linked polyubiquitin chain of E2F6, resulting in transcriptional repression of the phosphatase DUSP1. In addition, the inhibition of DUSP1 by E2F6 enhances the activation of AKT in HCC cells (84). USP22-E2F6-DUSP1-AKT axis promotes tumor growth and is expected to become a target for the treatment of liver cancer. Noncoding RNAs can play an important role in liver cancer by regulating USP22. LncRNA HULC can upregulate USP22, which, in turn, removes the polyubiquitin chain on COX-2. USP22 stabilizes COX2 protein, thereby promoting the proliferation of hepatoma cells (85).

### Other Tumors

USP22 plays an important role in the proliferation, differentiation, and cycle of breast cancer. USP22 promotes the deubiquitination of c-MYC in breast cancer cells, resulting in an increase in c-MYC level (65). USP22-mediated deubiquitination of c-MYC is closely associated with breast cancer progression. Furthermore, overexpression of USP22 stimulates breast cancer cell proliferation and aggregation and increases c-MYC tumorigenic activity (65). HSP90AB1, a target gene of USP22, is associated with poor prognosis in breast cancer. Loss of USP22 results in increased sensitivity of breast cancer to HSP90 inhibitors (104). Abnormal activation of the estrogen receptor alpha (ERα) signaling pathway promotes the malignant progression of breast cancer and promotes tumor resistance to endocrine therapy. USP22 inhibits ERα degradation by removing K48- and K63-linked ubiquitin chains of Erα (86). In addition, USP22 has been shown to inhibit unfolded protein response activity in human epidermal growth factor receptor 2 (HER2)-driven breast cancer (HER2-BC) cells by stabilizing the major endoplasmic reticulum chaperone HSPA5 (105). USP22 may be one of the major factors in breast cancer progression and a potential therapeutic target for endocrine resistance.

During glioblastoma development, USP22 exerts a tumor-promoting role through KDM1A deubiquitination (87). In gastric cancer–promoted progression, USP22 regulates FOXO1 and YAP signaling *via* c-MYC/NAMPT/SIRT1 (66). In addition, the USP22/SOS1/RAS axis is also a cancer-promoting pathway in gastric cancer (106). In PDAC, USP22 accelerates cancer cell proliferation by targeting DYRK1A (88). In retinoblastoma, USP22 depletion induces cancer cell apoptosis by inhibiting the TERT/P53 signaling pathway (89). In addition, USP22 is also closely related to the development of cancers such as nasopharyngeal carcinoma, oral squamous cell carcinoma, and thyroid carcinoma (107–109). In summary, USP22 downregulation reduces cancer cell proliferation, migration, and invasion and reduces tumor growth and metastasis *in vivo*. These studies suggest that USP22 may play an important role in the promotion of cancer, so it is expected to be a therapeutic target for preventing cancer progression.

In addition to playing a role in a variety of solid tumors, USP22 also has an important impact on hematological diseases. In most cancers, USP22 is considered an oncogene. However, in myeloproliferative neoplasms (MPNs) driven by oncogenic conditions Kras mutation, loss of USP22 results in myeloid leukemia. The specific mechanism of action is that the deletion of USP22 results in reduced levels of PU.1 under Kras mutation-driven MPN conditions, whereas previous studies have shown that reduced PU.1 expression and activity is common in mouse and human acute myeloid leukemia (AML) cases. Therefore, USP22 can promote the transformation of Kras mutant MPN into AML by affecting the stability and expression level of PU.1. This study provides new insights into the mechanism of USP22 transformation in MPN to AML (110).

## Conclusion and Future Perspectives

USP22 can exert oncogenic effects through multiple mechanisms and is associated with phenotypic changes that promote tumor development (111). USP22 is a key subunit of the SAGA complex that removes the ubiquitin chains of histones H2A and H2B. In addition to histones, USP22 deubiquitinates TRF1, CCNB1, CCND1, and SIRT1, thereby regulating involvement in metabolism, cycling, and apoptosis. USP22 plays a role in telomere maintenance by stabilizing TRF1 by deubiquitination. Recent studies (112) have shown that USP22 represses the transcription of the p21 gene by removing ubiquitin chains that regulate Lys 63 linkages on far upstream element–binding protein 1 (FBP1), leading to cell proliferation and tumorigenesis.

The role of USP22 in the tumor cell cycle cannot be ignored. Loss of USP22 resulted in cell cycle arrest in G1 phase and impaired MYC transcriptional function. USP22 deubiquitinates the G1 cyclin CCND1, thereby promoting the G1-S transition. USP22 affects p21 expression (P53 target gene) by altering FBP1 ubiquitination (112). USP22 can stabilize cyclin D1 and promote the nuclear accumulation of cyclin D1 (16). Because tumor is a disease of uncontrolled cell cycle and malignant proliferation, targeting USP22 to prevent cancer cell cycle and proliferation has great therapeutic potential.

The role of USP22 in the immune microenvironment may become an emerging hotspot. At present, there are few data, and the in-depth related mechanisms need to be further studied. Given that USP22 exhibits important roles in cancer development and drug resistance pathways, combination therapy of USP22 and other drugs has great potential. However, so far, there is no small-molecule inhibitor of USP22 that can be applied in clinic, which is an important issue for future research and clinical application.

Small-molecule inhibitors generally have the problem of low inhibition efficiency and easy to damage normal cells. These issues hinder drug development targeting USPs. USP22 is associated with a variety of signaling pathways and has a wide range of roles. Inhibition of USP22 may lead to extensive functional changes with unpredictable toxicity. Small-molecule inhibitors that inhibit USP22 are used in the clinic, and how to improve the specificity of their target substrates is a huge challenge.

It is important to develop a more specific inhibitor that inhibits some specific substrates. Our team is working with experts in molecular structure to improve the specificity of USP small-molecule inhibitors. We propose that the development of inhibitors based on the binding region of USP22 to downstream molecules can improve the specificity of its downstream action. In addition, USP22 inhibitors can be combined with targeted drugs through nanomaterials for precise delivery into tumors. Research targeting USP22 could be a promising strategy in cancer therapy.

## Author Contributions

(I) Conception and design: JG; (II) Administrative support: QX and DH; (III) Provision of study materials or patients: WF; (IV) Collection and assembly of data: JZ. All authors contributed to the article and approved the submitted version.

## Conflict of Interest

The authors declare that the research was conducted in the absence of any commercial or financial relationships that could be construed as a potential conflict of interest.

## Publisher’s Note

All claims expressed in this article are solely those of the authors and do not necessarily represent those of their affiliated organizations, or those of the publisher, the editors and the reviewers. Any product that may be evaluated in this article, or claim that may be made by its manufacturer, is not guaranteed or endorsed by the publisher.
